# P-532. Pediatric Perspectives of PrEP

**DOI:** 10.1093/ofid/ofae631.731

**Published:** 2025-01-29

**Authors:** Alex Rosencrance, Julia Rosebush, Jonathan Mannheim

**Affiliations:** University of Chicago Medicine, Chicago, Illinois; University of Chicago Medicine, Chicago, Illinois; University of Chicago Medicine, Chicago, Illinois

## Abstract

**Background:**

Thousands of teens are diagnosed annually with HIV-1 in the U.S. despite the 2018 approval of PrEP for adolescents by the FDA, highlighting the need for research on pediatric PrEP prescribing practices. Research in this area is scant and chiefly focuses on patient barriers to accessing PrEP. Our study explores barriers to prescribing for pediatric providers.

**Table 1:**
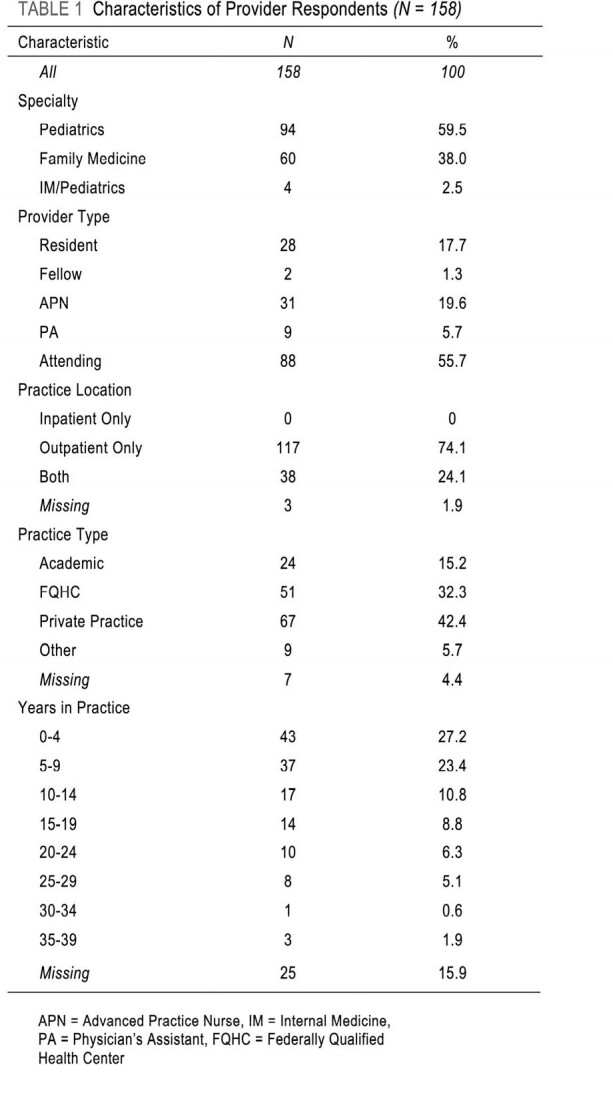
Characteristics of Provider Respondents

**Methods:**

We performed an online survey of medical providers from pediatrics, family medicine (FM) and internal medicine/pediatrics. A 5-point Likert scale (0-4) assessed comfort in providing adolescent sexual health care, including discussions around sexual activity, gender identity, sexual orientation, STIs, and PrEP. Potential barriers to PrEP prescription were explored. Unweighted proportions of responses were compared by provider characteristics, and logistic regression described the association between provider characteristics and the likelihood of having prescribed PrEP. Correlation matrix analysis identified strong relationships between item measure responses, identifying provider characteristics which associated with one another.

**Table 2:**
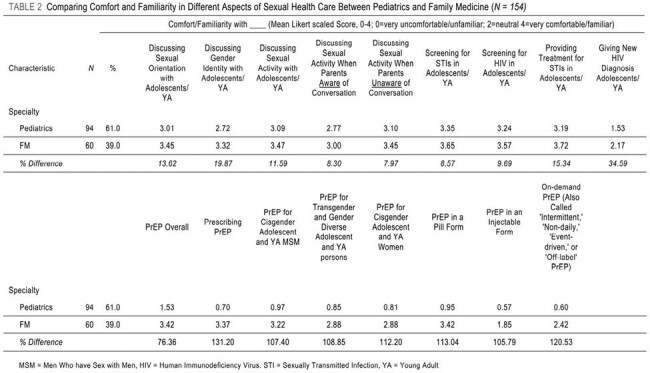
Comparing Comfort and Familiarity in Different Aspects of Sexual Health Care Between Pediatrics and Family Medicine

**Results:**

158 providers responded. Comparison of pediatric and FM providers found large differences in comfort with sexual health care. FM providers were more familiar with PrEP overall (3.42 to 1.53, 76.4% difference) and prescribing PrEP (3.37 to 0.70, 131.2%). FM providers were also more comfortable discussing sexual orientation (3.45 to 3.01, 13.62%), gender identity (3.32 to 2.72, 19.87%) and providing treatment for STIs (3.72 to 3.19, 15.34%). Logistic regression found that FM providers were 32 times more likely to prescribe PrEP than pediatric providers. Correlation analysis revealed that parental knowledge of a provider-patient conversation about sex decreased provider comfort in discussing sexual orientation and gender identity.

**Table 3:**
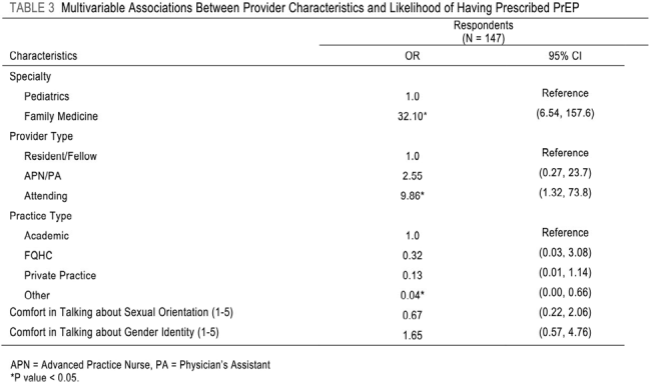
Multivariable Associations Between Provider Characteristics and Likelihood of Having Prescribed PrEP

**Conclusion:**

Pediatric providers were less comfortable than FM providers in matters related to sexual health, sexual identity, and gender identity, including PrEP. FM providers, who routinely see adults, were significantly more likely to prescribe PrEP than pediatric providers. Parental knowledge of patient-provider discussions of sex may decrease provider comfort with discussions about sexual orientation and gender identity.

**Table 4:**
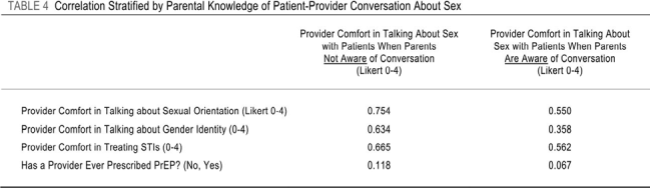
Correlation Stratified by Parental Knowledge of Patient-Provider Conversation About Sex

**Disclosures:**

**All Authors**: No reported disclosures

